# Whole genome and transcriptome reveal flavone accumulation in *Scutellaria baicalensis* roots

**DOI:** 10.3389/fpls.2022.1000469

**Published:** 2022-10-17

**Authors:** Suying Hu, Donghao Wang, Wentao Wang, Caijuan Zhang, Yunyun Li, Yueyue Wang, Wen Zhou, Junfeng Niu, Shiqiang Wang, Yi Qiang, Xiaoyan Cao, Zhezhi Wang

**Affiliations:** ^1^ National Engineering Laboratory for Resource Development of Endangered Crude Drugs in Northwest China, Key Laboratory of the Ministry of Education for Medicinal Resources and Natural Pharmaceutical Chemistry, Shaanxi Normal University, Xi’an, China; ^2^ Key Laboratory of Plant Resources Conservation and Sustainable Utilization, South China Botanical Garden, Chinese Academy of Sciences, Guangzhou, China; ^3^ University of Chinese Academy of Sciences, Beijing, China

**Keywords:** *Scutellaria baicalensis*, whole genome, transcriptomic, flavonoid biosynthesis, *S.baiMYB*

## Abstract

*Scutellaria baicalensis* Georgi is a medicinal plant in the Lamiaceae family that contains high levels of 4’-deoxyflavone and other flavonoids in its roots. Therefore, it has strong potential as a plant resource for researching the biosynthesis of specific flavonoids. In this study, we report on a chromosome-level *S. baicalensis* genome assembled to nine chromosomes (376.81M) using PacBio, HiSeq XTen, and Hi-C assisted assembly. The assembly ratio was 99.22%, the contig N50 was 1.80 million bases, and the scaffold N50 was 40.57 million bases, with 31896 genes being annotated. Comparative genome analysis revealed that *S. baicalensis* and *Salvia miltiorrhiza* belonged to the same branch, and diverged 36.3 million years ago. Other typically correlated species were *Boea hygrometrica* and *Sesamum indicum*. We investigated the structural genes involved in flavonoid synthesis in combination with transcriptome sequencing analysis for different tissues (roots, stems, flowers, leaves) of purple, pink, and white flowers. The results revealed that *S.baiF6H* is involved in the accumulation of baicalein and was significantly increased in both purple roots vs. pink roots and white roots vs. pink roots. *S.baiMYB* gene family expression pattern analysis and co-expression network analysis revealed that *S.baiMYB* transcription factors primarily regulated the production of flavonoids in *S. baicalensis*. *S.baiMYB* serves as a major factor regulating flavonoid synthesis in the roots, where yeast one-hybrid assays revealed that these transcription factors could bind to the promoter regions of structural genes to control the accumulation of flavonoids. Genome and transcriptome sequencing, co-expression analysis, and yeast one-hybrid experiments provided valuable genetic resources for understanding flavonoid biosynthesis in *S. baicalensis*. These findings contribute to a better understanding of the accumulation of metabolites in Lamiaceae.

## Introduction

*Scutellaria baicalensis* Georgi is a plant in the Lamiaceae family, and typically has purple flowers with bulbous roots that efficiently accumulate flavonoids. Its dried roots are called Huang-Qin (黄芩), which are primarily used to treat respiratory infections, diarrhea, dysentery, and liver disease in traditional Chinese medicine (Han et al., 2007). Its main active components consist of flavonoids and melatonin in the roots, of which baicalin comprises ~10–20%, and baicalin, baicalein, wogonin, and wogonin are the main active components (Bai et al., 2020). Its principal baicalin component has antibacterial, antiviral, anti-inflammatory, antioxidative, antitumor, neuroprotective, anticonvulsant, and cardiovascular protection properties that increase bone content, as well as anti-hyperglycemic properties (Zhao et al., 2016a).

In recent years, the structural genes of the flavonoid metabolic pathway have been investigated based on the *S. baicalensis* genome sequence (Zhao et al., 2019; Xu et al., 2020). Its flavonoid synthesis involved two pathways (classical flavonoid synthesis in the aerial components, and root-specific flavonoid 4’-deoxyflavonoid synthesis). The aerial flavonoid component is synthesized from an initial phenylalanine substrate *via* phenylalanine ammonia lyase (PAL), cinnamic acid 4-hydroxylase (C4H), 4-coumaric acid coenzyme A ligase (4CL), and 4-Coumaroyl coenzyme A (CoA). Subsequently, a 4-coumaroyl coenzyme A and three malonyl coenzyme A through the chalcone synthase (CHS) and chalcone isomerase (CHI) conversion of naringenin chalcone to naringenin. Flavonoid synthase (FNSII) synthesizes apigenin, after which flavonoid 6-hydroxylase is converted to scutellarin. Root-specific flavonoids are converted from phenylalanine through phenylalanine ammonia lyase (PAL) and cinnamate-CoA ligase (CLL-7) to cinnamoyl-CoA, whereas 4’-deoxyflavonoids are synthesized from cinnamoyl Coenzyme A, and malonyl coenzyme A are converted to pine chalcone by chalcone synthase (CHS) and chalcone isomerase (CHI). Next, chrysin is synthesized by flavonoid synthase (FNSII), and finally, flavonoid 6 -Hydroxylase (F6H) and flavonoid 8-hydroxyl (F8H) enzymes are converted to scutellarin and wogonin (Pei et al., 2022). The final flavonoids undergo methylation and glycosylation modification to generate stable flavonoids. Among them, the function of F6H is well described. Contrasting with the accumulation patterns of baicalin and the expression patterns of *S.baiF6H*, Which involved in the accumulation of baicalein in root of *S. baicalensis.* Zhao etc. reported the isolation and characterization of two *S.baiF6H* (SbCYP82D1.1 and SbCYP82D2) from *S. baicalensis*, which were able to convert chrysin to baicalein. When *S.baiF6H* was knocked down in *S. baicalensis* hairy roots, the content of baicalin was significantly reduced (Zhao et al., 2018). Liu etc. identified a F6H genes (CYP706X) from *Erigeron breviscapus* genome (Li et al., 2018). Xu etc. confirmed that seven F6H (CYP82D) genes could perform flavonoid 6-hydroxylase (F6H) catalytic activity in *S. baicalensis* and *S. barbata* (Gao et al., 2022).

The release of plant genome sequences has gradually expanded the genome of the Lamiaceae family of plants. Although the flavonoid metabolic pathway has been analyzed, the comparative genomes of relative flavonoid synthesis pathways in *S. baicalensis* have not been investigated and the regulatory genes involved in high-content flavonoid synthesis pathways have not yet been explored. The flavonoid biosynthetic pathway is regulated by the conserved MYB -bHLH - WD40 (MBW) complex in plants (Xu et al., 2015). *PAP1* (*AtMYB75*) and *PAP2* (*AtMYB90*) are positive regulators of flavonoid synthesis in *Arabidopsis* (Maier et al., 2013), while *AtMYBL2* and *AtMYB4* are negative regulators (Jin et al., 2000; Matsui et al., 2008). Previous research has demonstrated that *AtMYB113* can regulate flavonoid synthesis in *S. baicalensis via* regulatory pathways that determine their efficient accumulation (Yuan et al., 2013; Qi et al., 2015). For this study, whole-genome sequencing and transcriptome data, gene co-expression network analysis, and a yeast one-hybrid test were combined to reveal that *S.baiMYB* spatiotemporally regulated the flavonoid synthesis process, which provides evidence for the efficient accumulation of flavonoids in *S. baicalensis*.

## Materials and methods

### Plant materials and growth conditions

Purple-, pink-, and white-flowered *S. baicalensis* were planted in the resource garden of Shaanxi Normal University. Genome sequencing and transcriptome materials were extracted from the same plants at the same time. The purple-, pink-, and white-flowered *S. baicalensis* were grown during their second year, and transcriptome sequencing was performed at the flowering stage.

### PacBio genome sequencing and illumina sequencing

The genomic DNA of *S. baicalensis* was extracted using the CTAB method (Tel-zur et al., 1999); agarose electrophoresis was used to detect the DNA integrity; Nanodrop was used to detect the DNA purity and concentration, and Qubit was accurately quantified. A Covarisg-TUBE was used for fragmentation and magnetic beads were enriched for large DNA fragments. All samples passed quality control, fault repair, end repair, stem-loop junction, and PacBio sequencing (Hackl et al., 2014). Following the removal of low-quality reads and adapter sequences, the clear data volume, quality, and length were qualified for subsequent assembly analysis.

### Estimation of genome size

A survey was used to estimate the genome size, heterozygosity, and repeat sequences (Li et al., 2010). Third-generation assembly was conducted using 41 Gb subreads. All PacBio reads were first assembled using MECAT and Canu software and the resulting contigs were optimized using Quickmerge software (Boetzer and Pirovano, 2014); BUSCO was used to evaluate the assembly results data (Simao et al., 2015).

### *De Novo* assembly

The cross-linking of young tissues was performed using formaldehyde. Once the chromosomes were extracted and qualified, the chromatin was digested *via* restriction endonuclease (HindIII/MboI), labeled with biotin, and subjected to blunt end ligation, Hi-C sample preparation, and DNA quality detection. After the Hi-C library was qualified, Illumina HiSeq X Ten was sequenced, the data was filtered, and high-quality reads were obtained, which were aligned with the genome. The restriction fragment interactions were analyzed and assembled in the chromosomes (Belton et al., 2012).

### Genome annotation

Genome annotation consists primarily of repetitively annotated sequences, as well as gene structure and gene function prediction. Sequences akin to known repeat sequences were identified in the RepBase repeat sequence database using RepeatMasker and RepeatProteinMask software (Price et al., 2005). A *de novo* repeat sequence library was constructed using the RepeatModeler software and predicted with the RepeatMasker software. Genes were aligned using BLAST, Augustus, GlimmerHMM, SNAP, and GeneMark software, as well as the statistical characteristics of genome sequence data (Chaisson and Tesler, 2012). The gene structure was predicted using the EVidenceModeler method and was non-redundantly integrated. Functional gene annotation was derived from gene structural annotation and compared using various functional databases, including SwissProt, not, nr, Pfam, eggNOG, GO, and KEGG databases. Two strategies were used for the annotation of ncRNAs: the Rfam database to compare rRNAs and snRNAs, and miRNAs with known non-coding RNA libraries. The tRNA genomic sequences were predicted using tRNAs can-SE.

### Evolutionary analysis

Gene family cluster analysis: Gene candidate species were filtered, which included several variants spliced transcripts of a gene, and only transcripts with the longest coding regions were retained for further research (Parra et al., 2007). To ensure the efficacy of protein encoding, genes that encoded proteins with fewer than 50 amino acids were excluded. BLASTp was used to detect similarities between protein sequences from all species, where the e-value was set to 1e-5 by default. OrthoMCL software was used to cluster the results, with an expansion ratio of 1:5. The phylogenetic analysis of 567 single-copy orthologous gene families, MUSCLE1 (http://www.drive5.com/muscle/) alignment for each family, and alignment results were merged to generate a super alignment matrix. A 10-species (ML TREE) was constructed *via* 4d locus and PhyML software using the maximum likelihood method. Phylogenetic trees are typically developed using BRMC.via cmctree in the PAML software package (http://abacus.gene.ucl.ac.uk/software/paml.html). Gene families with abnormal gene numbers in individual species were filtered using the PGM (probabilistic graphical models) model in CAFE software (http://sourceforge.net/projects/cafehahnlab/). Gene gains and losses were simulated in the defined evolutionary tree, and gene family growth and contraction were analyzed using hypothesis testing. The MCscan program used Musclesoftware (http://chibba.agtec.uga.edu/duplication/mcscan/) to achieve multiple sequence alignment of the sequences within the block. The 4dTV value was then calculated and the overall species occurrence was estimated, depending on the abundance of the 4dTV value. The the genome and interspecies differentiation were duplicated.

### RNA sequencing

Six tissues were harvested from *S. baicalensis*, namely flower, leaves, purple flower *S. baicalensis* of roots, stems, pink flower *S. baicalensis* of roots, white flower *S. baicalensis* of roots. Three biological replicates for each tissue were collected. The total RNA was extracted using phenol/chloroform. After qualifying the total RNA samples, magnetic beads bearing Oligo (dT) were used for enrichment. A polyA tail RNA was found at the 3’ end of the mRNA. Polymerase I was used to create the second strand of cDNA, which was then purified using the QIAQuick PCR kit and eluted with an EB buffer. To complete the library preparation, the purified double-stranded cDNA was exposed to end repair, base A, and sequencing adapters, after which the target size fragments were recovered by agarose gel electrophoresis and PCR amplification. Following the construction of the library, Qubit3.0 was used for preliminary quantification, and Agilent 2100 was used to identify the insert size of the library. Quantitative PCR was performed using Bio-rad CFX 96. The Illumina X Ten platform was used to sequence the qualified libraries. The raw reads obtained from Illumina sequencing were processed to obtain high-quality sequences (Clean Reads) by eliminating low-quality sequences and decontaminating the adapters. Clean reads were aligned to our *de novo* genome of *S. baicalensis* using TopHat (Trapnell et al., 2012). Reads provided the foundation for all future assessments and compared the differentially expressed genes at various thresholds. We selected |log2 fold-change (FC)| ≥1, where pvalue <0.05 was considered significantly different. The DEGs were annotated using blast NCBI, Uniprot, GO, and KEGG databases, which were performed by hierarchical and *K*-means clustering. A hypergeometric test was used to identify the highly enriched pathways in differentially expressed genes to determine whether the pathways were considerably enriched (Mortazavi et al., 2008), Annotation of transcript datasets in different tissues (roots, stems, flowers, leaves) of purple, pink roots and white roots with FPKM are listed in **Supplementary Tables S2**, **S3**.

### Phylogenetic analysis

Multiple sequence alignments were performed using ClustalW alignment (Larkin et al., 2007). The phylogenetic tree was designed using MEGA10 (NJ, 1,000 boots) (Tamura et al., 2013). 1.5 kb promoter fragments of structural genes were extracted and queried against PlantCARE. TF binding sites were illustrated using TBtools. *S. baiMYB*s sequences are given in **Supplementary Table S4**

### Coexpression analysis

Correlation analyses between structural genes and *S.baiMYB* involved in flavonoid synthesis with correlation coefficients > 0.8 were performed separately using advanced correlation tetwork (pearson, p<0.05) (Metware Cloud). Detailed samples are included in **Supplementary Table S5**.

### Yeast one-hybrid

The promoter sequences of structural genes were searched for the genome. The promotors were inserted into the pHis2-Leu-GW vector using the In-Fusion method, and the *S.baiMYBs* gene was digested and ligated into the pGADT7 vector. The vectors were mixed to transform Saccharomyces cerevisiae Y187. Transformants were cultured in SD/-Leu/-Trp/-His medium and SD/-Leu/-Trp/-His + 60 mM 3-AT medium for 3 days, respectively. p53-his2 was transformed as a negative control, while p53-his2 and pGADT7-p53 were used as positive controls. The primer sequences are displayed in **Supplementary Table S6**.

### Statistics

All experiments were performed with three biological replicates unless otherwise specified. The data were the average of three technical repetitions expressed as mean ± standard error. ANOVA analysis was used for statistical analysis; the probability value P < 0.05 was considered statistically significant.

## Results

### Whole-genome sequencing data of *S. baicalensis*

**Figure 1:**
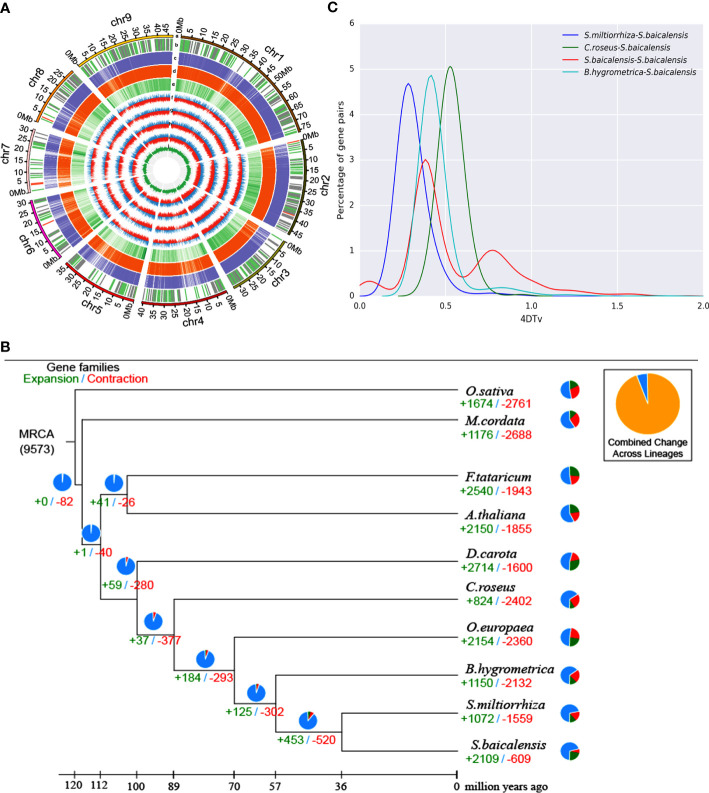
Genome and evolutionary analysis of *S. baicalensis.* **(A)** Overview of *S. baicalensis* genome assembly and annotation. (a) chromosome number and chromosome length; (b) non-coding RNA, rRNA, tRNA, and other types of non-coding RNA were distinguished on the genome (red, green, and grey, respectively); (c, d) Homologous gene data for *Salvia miltiorrhiza* and *Arabidopsis thaliana*; (e) Gene abundance (f-i) transcriptome of the expression data of flowers, leaves, stems and roots; (j) GC content. **(B)** Gene family expansion and contraction. Pie chart representing the proportion of gene expansion and contraction. **(C)** Whole-genome duplication analysis.

The entire nuclear *S. baicalensis* genome was sequenced using HiSeq X Ten, PacBio, and Hi-C assisted genome assembly, and the sequencing data of two other researchers were summarized (**Figure S1**). We obtained a genome of 376.81M in size, with a 99.22% assembly rate assembled into nine chromosomes (**Figure 1A**). The ScaffoldN50 was 40.57 million bases and the ContigN50 was 1.80 million bases. Genome annotation revealed that the *S. baicalensis* genome was 57.73% repetitive, which presents a chromosome-scale genome assembly for *S. baicalensis* with longer reads and coverage (**Table 1**). *Salvia miltiorrhiza*, *Arabidopsis thaliana*, and *Sesamum indicum* were used as reference genes for structural prediction. There were 31,896 gene annotations, with an average gene length of 2605.09 bp in *S. baicalensis* (**Supplementary Table S1**). An analysis of gene families in 15 closely related species of *S. miltiorrhiza*, and *S. indicum* and external species included *Olea europaea*, *Catharanthus roseus*, *Boea hygrometrica*, *Arabidopsis thaliana*, *Daucus carota*, outer species *Oryza sativa*, *Fagopyrum tataricum*, and *Macleaya cordata*, comprising 567 single-copy gene families of 25,706 clustered genes (**Figure 1B**). Venn diagram analysis selected 10 species, of which six gene families from the studied species were functionally annotated *via* phylogenetic analysis (**Figure S2**). The results revealed that *S. baicalensis* and *Salvia miltiorrhiza* belonged to one branch, whereas *S. baicalensis* and *S. miltiorrhiza* were closely related to each other. The divergence time was 36.3 million years ago (Mya) for *S. baicalensis* and *S. miltiorrhiza*, while for *B. hygrometrica* it was 57 Mya (**Figure S3**). *S. baicalensis* had a 2,109 gene family expansion and 609 gene family contraction comparison reference genome (**Figure 1B**). There was a 373 gene family expansion and 40 gene family contraction with significant differences in the phylogenetic tree. 567 single-copy paralogous gene pairs were investigated whole-genome duplication (WGD) events during *S. baicalensis* evolution. The Ks values showed genome duplication events at 0.36 and 0.74 in *S. baicalensis.* The genome data of *S. miltiorrhiza, B. hygrometrica and C. roseus* were represened species specific or different plant species, which analysised recent WGD event. The results showed that the Ks values peaked at approximately 0.25, 0.38 and 0.51 in *S. miltiorrhiza, B. hygrometrica and C. roseus.* The distribution of the Ks values showed that a WGD events have occurred before the divergence of *B. hygrometrica* and *C. roseus*. There is a WGD events have occurred since the divergence of *S. miltiorrhiza* (**Figure 1C**).

**Table 1:** Statistics for genome sequencing of *S. baicalensis*.

Assembly feature	Qing Zhao etc.	Zhichao Xu etc.	Suying Hu etc.
Genome Size (Mbp)	408.14 Mb	441,861,814	365.92 Mb
Genome Length (bp)	386.63 Mb	376,971,275	376,808,447
ContigN50	880,642	2,102,880	1,803,970
Scaffold	386,627,338		376,862,547
ScaffoldN50	33.2 Mb	40,790,749	40,573,676
Max	87.96 Mb		76,118,278
Map rate (%)	96.50%	88.72%	99.22%
Complete BUSCos (C)	C: 64% [D: 22%], F: 1.7%, M: 3.4%, n:956	C:91.5%, F: 2.6%, M:5.9%, n: 1440	C: 90.2% [D: 11.7%], F: 2.6%, M: 2.8%, n: 1440
No. of predicted transcripts and proteins	28930	31896	33414
Average gene length (bp)	2,980.36	4553	2605
Average CDS length (bp)	1112.48	1130	1068
Hi-C assembly N50	33.20 Mb	40.8 Mb	40.57Mb

### Transcriptome analysis of *S. baicalensis*

**Figure 2:**
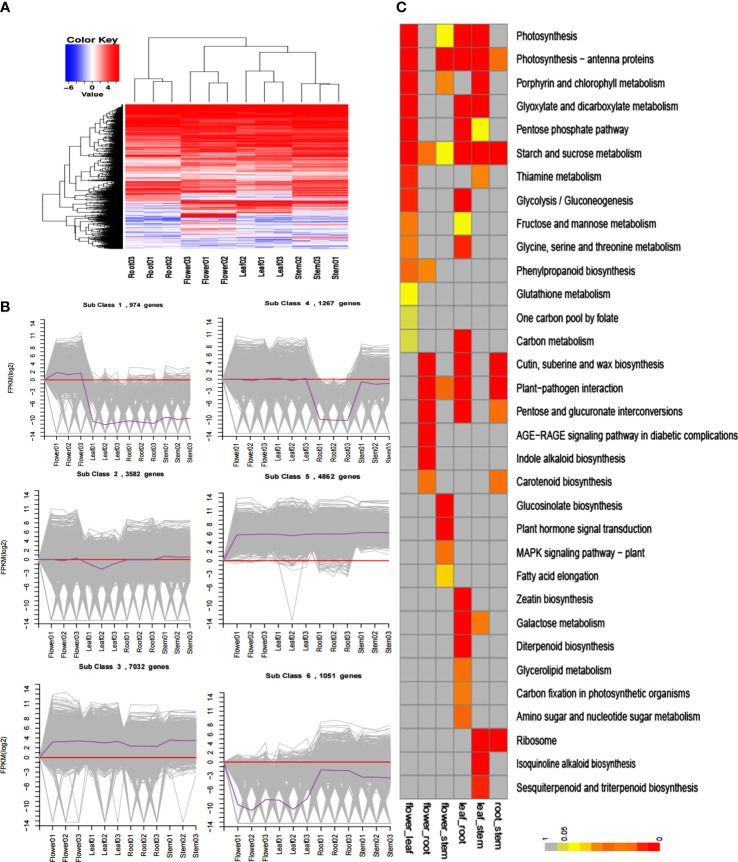
Transcriptome analysis of *S. baicalensis* roots, stems, flowers, and leaves. **(A)** Hierarchical cluster of differentially expressed genes. **(B)** *K*-means cluster. Grey lines show the expression patterns of genes in each cluster, and dark purple lines indicate that all genes in the cluster are in the sample. **(C)** KEGG enrichment. Different colors represent different levels of enrichment.

A total of 56 million clean reads were obtained through Illumina X Ten sequencing, after which comparative and expression analyses were performed by hierarchical and *K*-means clustering (**Figures 2A, B**). The comparative expression patterns were significantly different between the roots, stems, flowers, and leaves. The DEGs were analyzed using KEGG Enrichment, and the phenylpropane synthesis pathways and terpenoids were significantly different between the four tissues (**Figure 2C**).

### Structural gene analyses of flavonoid biosynthesis pathways combined with transcriptome in *S. baicalensis*

**Figure 3:**
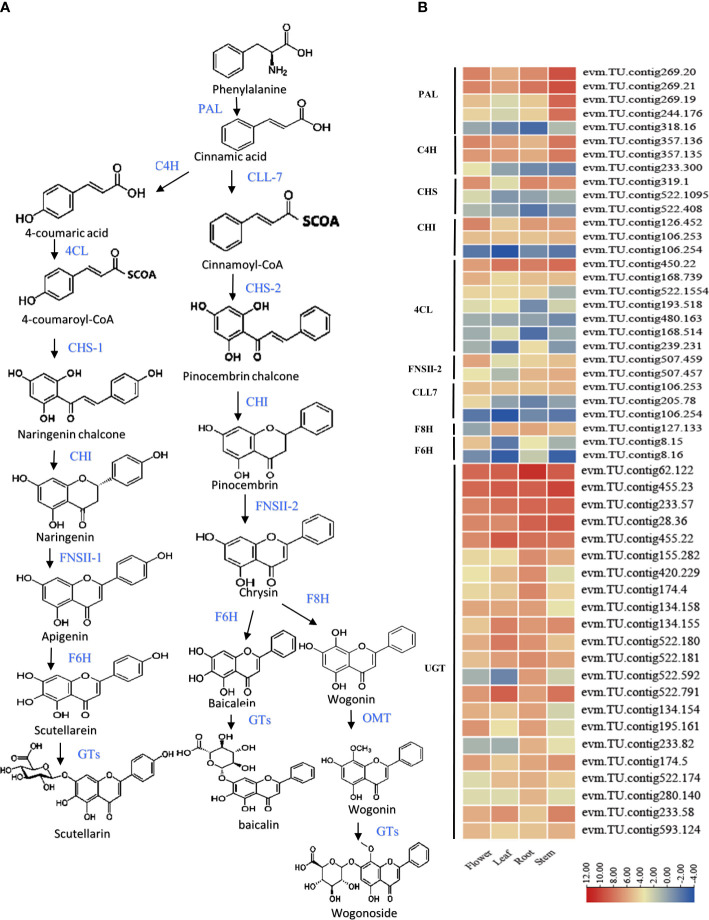
Synthesis pathway of flavonoids in *S. baicalensis*. **(A)** Flavonoid biosynthesis pathway structural genes including phenylalanine ammonia lyase (*S.baiPAL*), cinnamate 4-hydroxylase (*S.baiC4H*), cinnamate-CoA ligase (*S.baiCLL-7*), 4-coumarate CoA ligase (*S.baiCLL-1*), chalcone synthase (*S.baiCHS-1*), pinocembrin- chalcone synthase (*S.baiCHS-2*), chalcone isomerase (*S.baiCHI*), flavone synthase II (*S.baiFNSII*), flavone 6-hydroxylase (*S.baiF6H*), flavone 8-hydroxylase (*S.baiF8H)*, and 8-O-methyl transferase (*S.baiOMT*). **(B)** Heatmap of structural gene expression in stems, leaves, and flowers. Low to high expression is indicated by changes in color, from blue to red. Three biological replicates were used for each sample.

The flavonoid biosynthesis expression patterns were summarized for *S. baicalensis*, and structural genes are displayed. (*S.baiPAL* (5), *S.baiC4H* (3), *S.bai4CL* (7), *S.baiCHS* (3), *S.baiFNSII* (2), *S.baiCHI* (3), *S.baiF6H* (2), *S.baiF8H* (1), and *S.baiUGT* (22)) (**Figure 3A**). Transcriptome data revealed that *S. baiF6H* was significantly more highly expressed in the roots of *S. baicalensis*, while the other structural genes were not specifically expressed in the four tissues (**Figure 3B**).

### Transcriptome analysis of three germplasms of *S. baicalensis*

**Figure 4:**
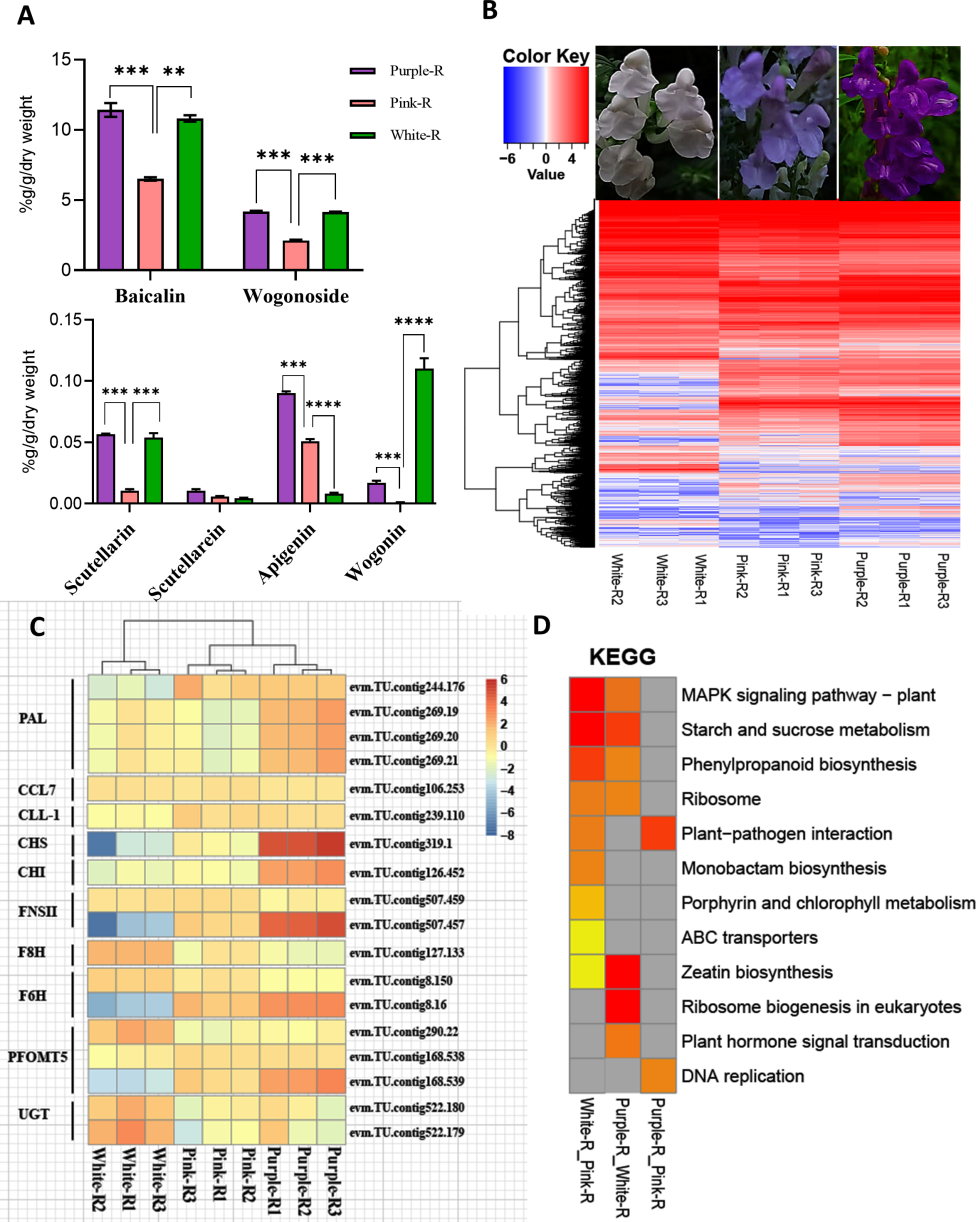
Transcriptome analysis of different germplasms of *S. baicalensis*. **(A)** Structural gene differential gene expression calorimetry. **(B)** KEGG clustering heatmap. **(C)** Histogram of baicalin, baicalein, wogonin, and wogonin contents. **(D)** Structural gene expression heatmap. Low to high expressions are indicated by changing colors, from blue to red. Data are the mean ± SD from three biological replicates. Asterisks (*) indicate significant differences (**P < 0.01, ***P < 0.001, ****P < 0.0001 based on Duncan’s multiple range test).

RNA-seq data identified DEGs in three germplasms of *S. baicalensis* (**Figure 4B**). The results showed that the structural genes of flavonoid biosynthesis significantly decreased in the white and pink roots (*S.baiCHS*, *S.baiCHI*, *S.baiFNSII*), while *S.baiF8H*, *S.baiF6H*, and *S.baiPFOMT* were upregulated in the white and purple roots (**Figure 4C**). The major bioactive chemical constituents (e.g., baicalin, wogonin, and scutellarin) were significantly higher in the *S. baicalensis* roots of the purple variety (**Figure 4A**). The DEGs were analyzed using KEGG enrichment, with the outcomes revealing that the phenylpropane synthesis pathways were significantly different between the three germplasms of *S. baicalensis* (**Figure 4D**).

### Identification *MYB* transcriptional regulatory network in *S. baicalensis*

**Figure 5:**
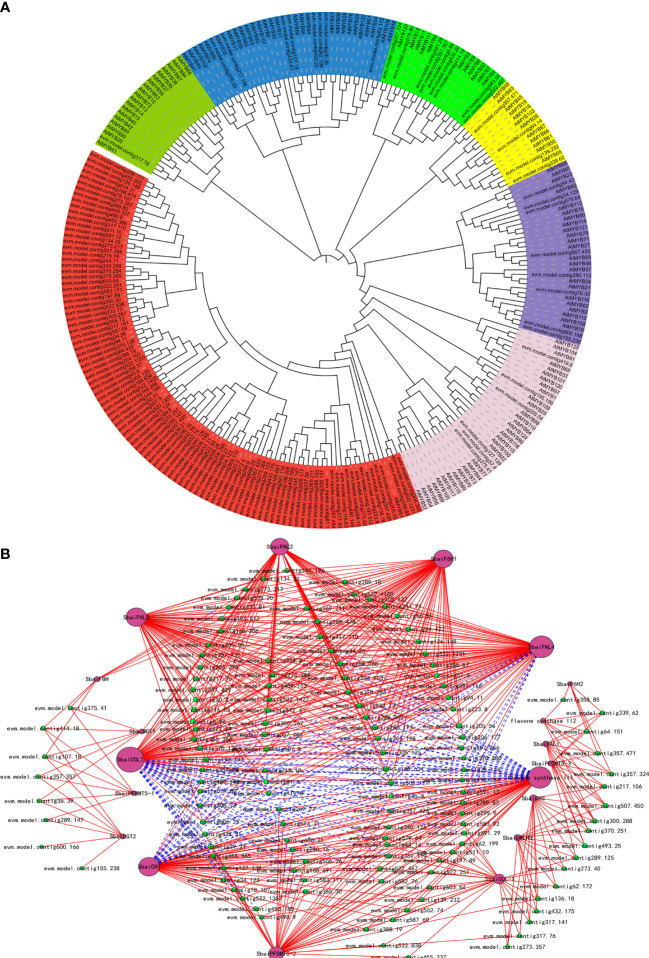
*S.baiMYB*s evolution and correlation analyses of structural genes. **(A)** *S.baiMYB*s and *AtMYB*s were used to construct a phylogenetic tree (neighbor-joining method). **(B)** *S.baiMYBs* and structural gene co-expression network analysis. The data are mean ± SD from three biological replicates.

A total of 134 *MYB* candidate genes were identified through the Pfam database of the *S. baicalensis* genome. The results indicated that the phylogenetic tree separated the *S. baicalensis MYB* members into 20 groups, and a large number of *S.baiMYB* groups were not included in the *A. thaliana* branch (**Figure 5A**). A co-expression analysis of *S.baiMYBs* and flavonoid structural genes was performed according to the *S. baicalensis* root transcriptome data for white, pink, and purple flowers (**Figure 5B**). The results showed that *S.baiMYBs* transcription factors were negatively correlated or positively correlated with *S.baiCHI*, *S.baiFNS*, and *S.baiUGT*. *S.baiMYBs* transcription factors were positively correlated with *S.baiF6H* and *S.baiF8H*. We selected 10 transcription factors related to flavonoid synthesis that were closely related to the *AtMYB* gene (e.g., *S.baiPAP1* (evm.model.contig94.43), *S.baiPAP2* (evm.model.contig34.129), *S.baiMYB111* (evm.model.contig357.324), *S.baiMYB116* (evm.model.contig76.33), *S.baiMYB70* (evm.model.contig57.4), *S.baiMYB60* (evm.TU.contig36.39), and *S.baiMYB5* (evm.TU.contig357.357) were positively correlated. *S.baiMYB3* (evm.model.contig535.20) and *S. baiMYB4* (evm.model.contig8.96) were negatively correlated. The prediction results showed that most MYBs could bind to the promoter regions of structural genes. However, *S.baiCLL-7*, *S.baiF6H*, and *S.baiF8H* had fewer binding sites (**Figure 5C**).

### Yeast one-hybrid analysis *MYB* transcription factor

**Figure 6:**
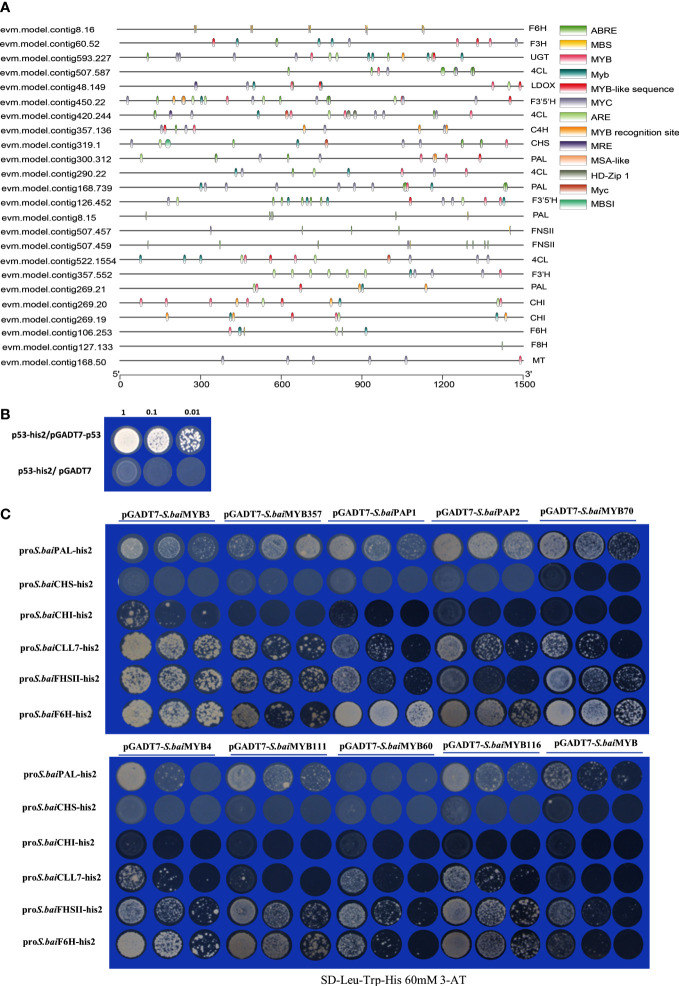
Yeast one-hybrid experiment. **(A)** Structural gene promoter region prediction. **(B)** pGADT7-p53 and p53-his2 are positive controls, and p53-his2 and pGADT7 are negative controls. **(C)** *S.baiMYB*s and structural gene promoter analysis.

Transcription factor binding site prediction results verified that most MYBs bind with sites in the promoter regions of structural genes (**Figure 6A**). P53 and pGADT7 were used as positive and negative controls (**Figure 6B**), respectively, for the yeast one-hybrid analysis of candidate *S.baiMYB* transcription factors and structural genes (**Figure 6C**). The yeast one-hybrid results revealed that fewer MYBs could bind to the promoter regions of structural genes (*S.baiCLL-7*, *S.baiF6H*, and *S.baiF8H*). *S.baiMYB3*, *S.baiMYB4*, *S.baiPAP1*, and *S.baiPAP2* could bind to *S.baiPAL*, *CLL-7*, *S.baiFNSII*, and *S.baiF6H*, as well as *S.baiCHS* and *S.baiCHI* promoters. Furthermore, *S.baiMYB60*, *S.baiMYB70*, and *S.baiMYB111* could be combined with *S.baiPAL*, *CLL-7*, *S.baiFNSII*, and *S.baiF6H* promoters.

## Discussion

As a popular Chinese herbal medicine, *S. baicalensis* has long been used in China (Zhao et al., 2016a). Traditional compound medicines such as Xiao Chai Hu Tang and Fei Fu Fang are extensively used in clinical practice. Xiao Chai Hu Tang has hepatoprotective effects, which were introduced to the alternative medicine community in the United States. These can increase chemotherapeutic effects for non-small cell lung cancers. Flavonoids (e.g., baicalin, baicalein, wogonin, and wogonin) are the primary active components in *S. baicalensis* (Bocho áková et al., 2003). Utilizing high-quality genome sequencing, we investigated the molecular synthesis and efficient accumulation mechanisms of unique metabolites. Medicinal plants of the Lamiaceae family, such as *Mentha longifolia, S. miltiorrhiza, Salvia bowleyana, Scutellaria barbata, Salvia splendens, Ocimum basilicum, Origanum majoran a, Origanum vulgare, Pogostemon cablin*, and *Rosmarinus officinalis* have been sequenced (Meng et al., 2022). Among them, *S. miltiorrhiza*, *S. bowleyana*, *S. barbat*, and *P. cablin* were assembled chromosomes. *S. baicalensis* genome evolution analysis revealed that *S. barbata* and *S. baicalensis* diverged about 70,000 years ago (Ming et al., 2018). *S. splendens*, *S. miltiorrhiza*, *S. bowleyana*, and *S. barbara* are closely evolutionarily related.

The medicinal components of *S. baicalensis* exist primarily in its roots (Wang et al., 2018). Zhao et al. found that the aboveground portion is mostly comprised of scutellarin, while the belowground portion is mostly made up of *S. baicalensis* flavonoids (Zhao et al., 2016b; Zhao et al., 2018). JA treatments have been shown to increase the expression of the structural genes of flavonoids in *S. baicalensis* (Yuan et al., 2013). However, the high-efficiency accumulation of flavonoids in the roots of *S. baicalensis* remains unknown, as do the expression patterns of the genes involved. Transcriptome data revealed that the high expression of *S. baiF6H* was positively correlated with the synthesis of baicalein flavonoids and their efficient accumulation in the roots.

The MYB-bHLH-WD40 (MBW) complex is a key factor in the regulation of flavonoids, with direct or indirect roles in regulating genes during the accumulation of plant flavonoids (Ramsay and Glover, 2005; Li, 2014). It is one of the largest gene families of *MYB* transcription factors in plants that directly regulate flavonoid synthesis. *AtMYB75/PAP1 (* Rowan et al., 2009 *)*, *AtMYB90/PAP2(* Li et al., 2018 *)*, *AtMYB5 (* Li et al., 2009 *)*, *AtMYB111 (* Stracke et al., 2010 *)*, *AtMYB113 (* Liu et al., 2016 *)*, and *AtMYB114 (* Yao et al., 2017 *)* (subfamily 6) positively regulate the biosynthesis of anthocyanin in *A. thaliana* and can regulate the expression of genes such as chalcone synthase (*CHS*), chalcone isomerase (*CHI*), and flavonol 3-hydroxylase (*F3H*). Flavonoid synthesis inhibitors include *AtMYB34*, *AtMYB29*, *AtMYB76*, *AtMYBx*, *AtMYBL1*, *AtMYB3L*, *AtMYB27*, *AtMYB3*, *AtMYB4*, *AtMYB7*, and *AtMYB32 (* Gates et al., 2018 *)*. The overexpression of *S.baiMYB* in tobacco can increase the accumulation of flavonoids (Qi et al., 2015). Most *S.baiMYB* in *S. baicalensis* and *AtMYB* are not in the same evolutionary clade, and there are also fewer repressors clustered within the same evolutionary clade.

We used co-expression network analysis based on transcriptome sequencing results from rhizome mosaic leaves and *S. baicalensis* roots to describe the structural genes of flavonoid synthesis in *S. baicalensis*. We discovered fewer transcriptional repressors of the flavonoid contracting process, while the majority of these *MYB*s served as positive regulators. *S. baiMYB3* and *S. baiMYB4*, which act as repressors, were expressed at high levels in flowers but low levels in roots. The high expression of repressive *S. baiMYBs* in the aboveground portion of the plant decreased the flavonoid synthesis in *S. baicalensis*, which was highly accumulated in the roots. It was observed *via* transcriptional expression analysis that the specific spatiotemporal presence of *S. baiMYBs* and structural genes were highly significant, which was essential for flavonoid accumulation. According to yeast one-hybrid experiments, positive regulatory transcription factors regulated the expression of *S. baiCLL-7*, *S. baiFNSII*, and *S. baiF6H* genes. As such, this study elucidated the main reasons behind the efficient accumulation of flavonoids in *S. baicalensis*.

## Conclusion

In this study, we reported the reference genome of *S. baicalensis*, while comparison with different tissues (roots, stems, flowers, leaves) of purple, pink, and white flowers revealed that F6H *S.baiF6H* is involved in the accumulation of baicalein in three germplasms of *S. baicalensis* roots. The *S.baiMYBs* gene family regulated the production of baicalein in *S. baicalensis* roots.

## Data availability statement

The original contributions presented in the study are publicly available. This data can be found here: https://ngdc.cncb.ac.cn/, PRJCA009554 & PRJCA009556.

## Author contributions

ZW conceived of and designed the project. SH, DW, and WW performed the experiments and analyzed the data. SH wrote the original draft of the paper. YL, YW, WZ, JN, SW, YQ, and XC revised the paper. All authors contributed to the article and approved the submitted version.

## Funding

This research was supported by the National Key Technologies R&D Program for Modernization of Traditional Chinese Medicine (2017YFC1701300), the Key Industry Chain Project of Shaanxi province (2022ZDLSF05-01), the National Natural Science Foundation of China (3217020611), the Central University Project (GK202003053), and the Fundamental Research Funds for the Central Universities (GK202205002, GK202205003, GK202205004,GK202205006).

## Conflict of interest

The authors declare that the research was conducted in the absence of any commercial or financial relationships that could be construed as a potential conflict of interest.

## Publisher’s note

All claims expressed in this article are solely those of the authors and do not necessarily represent those of their affiliated organizations, or those of the publisher, the editors and the reviewers. Any product that may be evaluated in this article, or claim that may be made by its manufacturer, is not guaranteed or endorsed by the publisher.

## Supplementary material

Supplementary Figure 1 Interaction heat-map of chromosomal fragments based on Hi-C analysis.

Supplementary Figure 2 Common and unique gene families based on Venn diagram analysis.

Supplementary Figure 3 Estimation of divergence time analysis.
