# Supplementary figures and images for: Whole genome and transcriptome reveal flavone accumulation in Scutellaria baicalensis roots
Source: Front Plant Sci. 2022 Oct 17;13:1000469. doi: 10.3389/fpls.2022.1000469 (PMC9618734; doi:10.3389/fpls.2022.1000469)

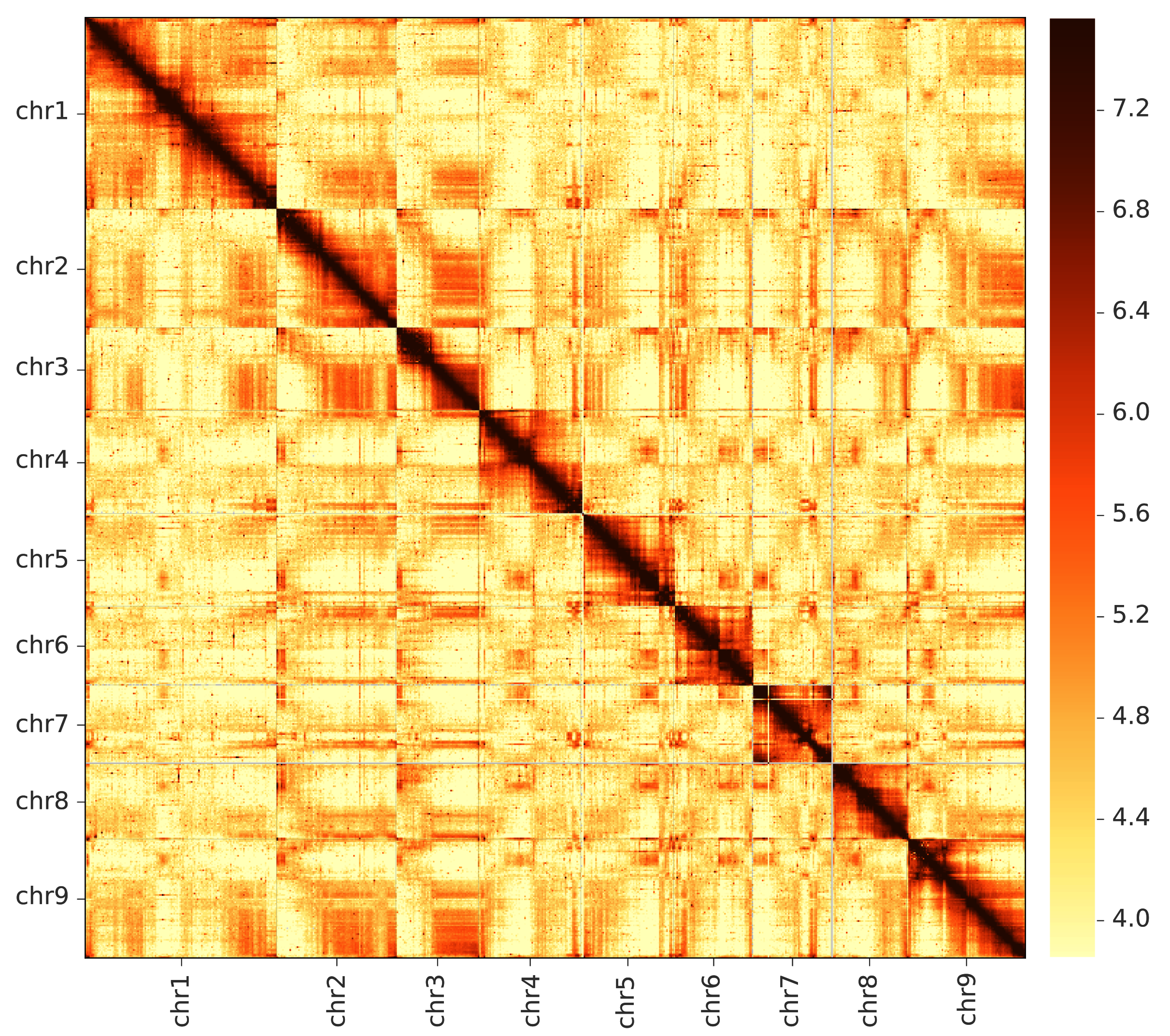

Supplement: Supplementary Figure 1 — Interaction heat-map of chromosomal fragments based on Hi-C analysis. [file Image_1.tif]

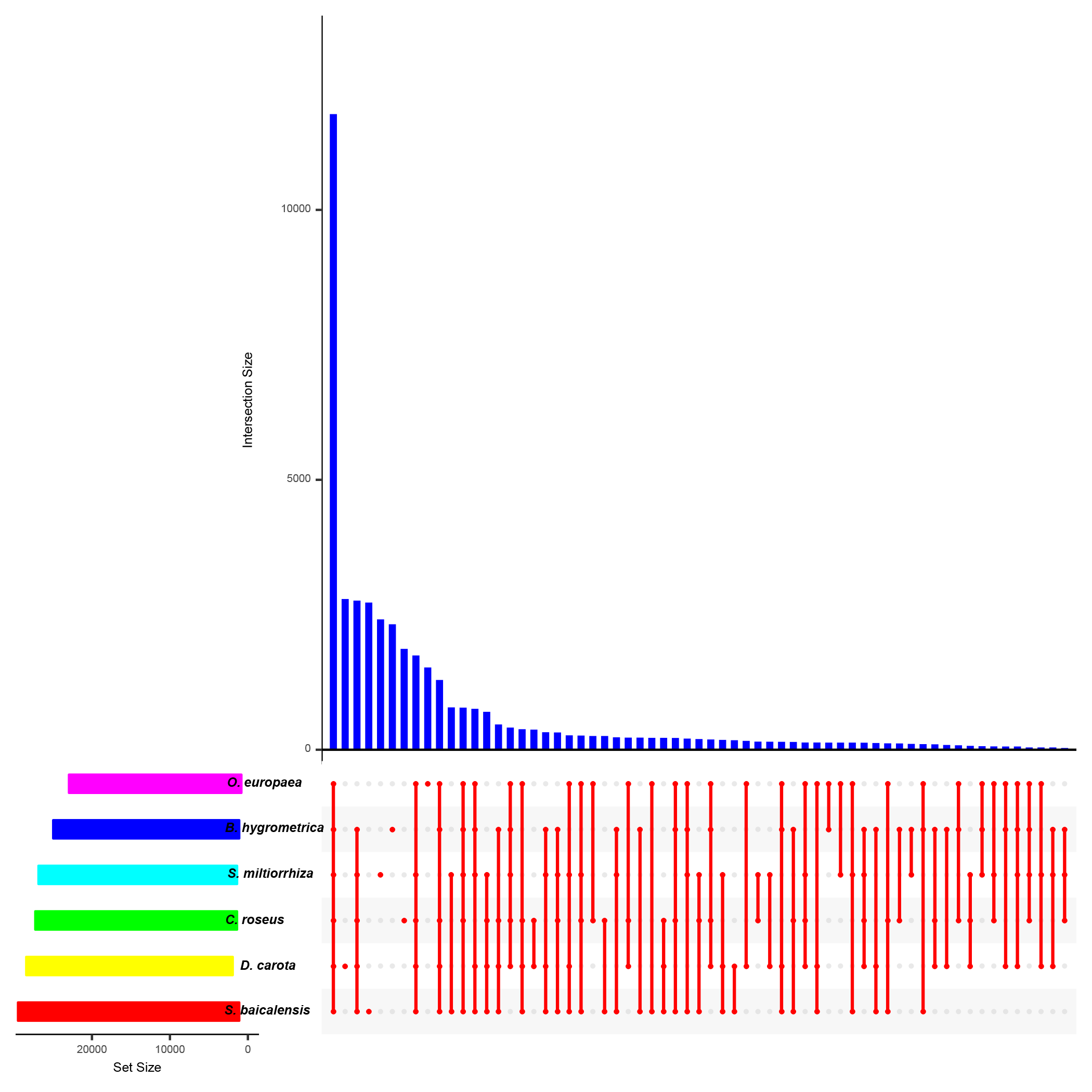

Supplement: Supplementary Figure 2 — Common and unique gene families based on Venn diagram analysis. [file Image_2.tif]

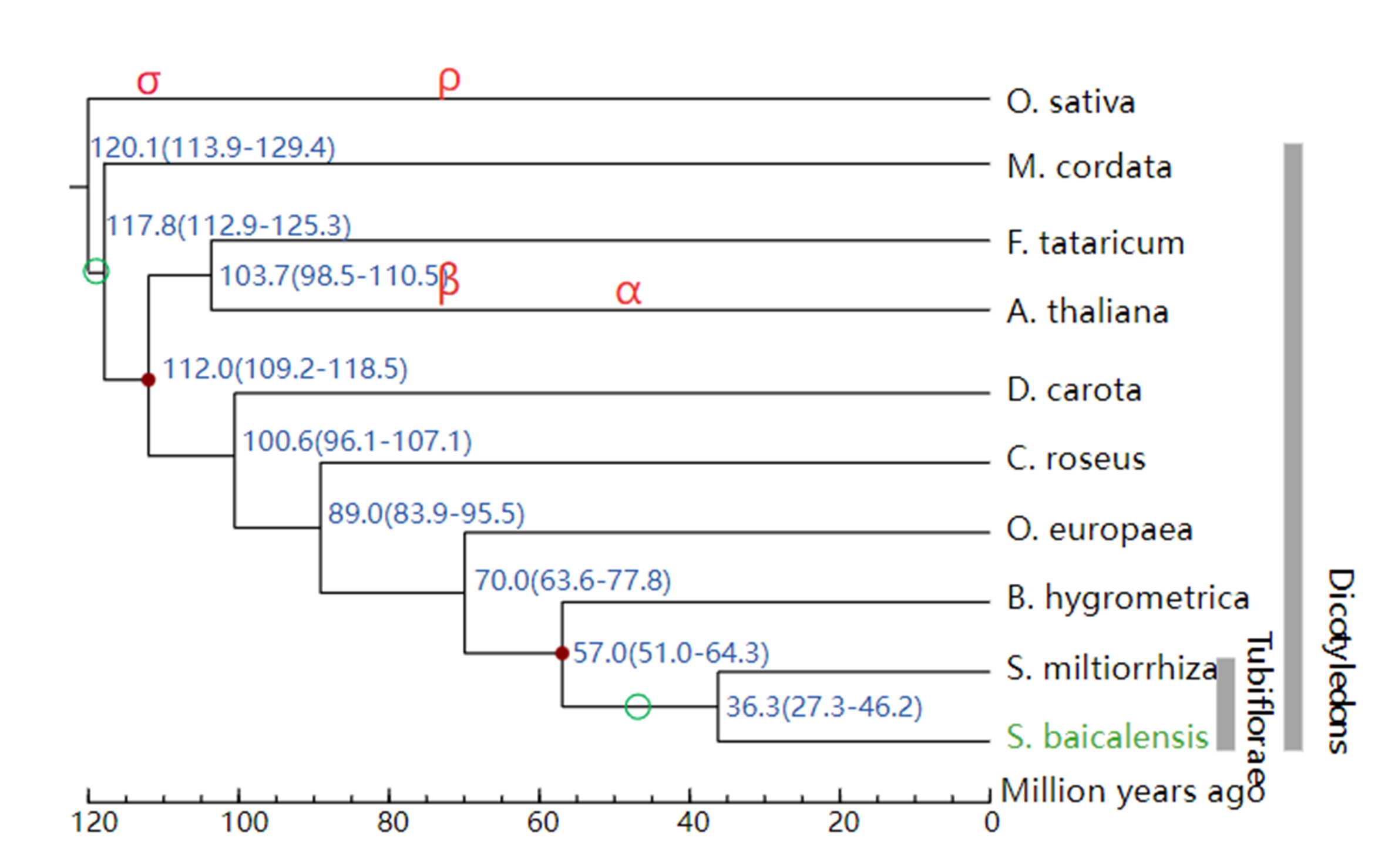

Supplement: Supplementary Figure 3 — Estimation of divergence time analysis. [file Image_3.tif]
